# RNA-seq and qRT-PCR analyses reveal the physiological response to acute hypoxia and reoxygenation in *Epinephelus coioides*

**DOI:** 10.3389/fphys.2022.1049776

**Published:** 2022-11-03

**Authors:** Xingxing Lai, Zhongxuan Zhong, Bing Lin, Yuxin Wu, Yonghao Ma, Cuiping Zhang, Yang Yang, Mingqing Zhang, Weijian Qin, Xiaoqin Fu, Hu Shu

**Affiliations:** ^1^ School of Life Sciences, Guangzhou University, Guangzhou, China; ^2^ School of Life Sciences, Sun Yat-sen University, Guangzhou, China

**Keywords:** Epinephelus coioides, hypoxia stress, HIF-1 signaling pathway, transcriptome, qRT-PCR, HIF-1α

## Abstract

Hypoxia is a critical problem in intensive *Epinephelus coioides* aquaculture systems. In the present study, the physiological responses of *E. coioides* muscle to acute hypoxic stress (DO = 0.6 ± 0.1 mg/L) and reoxygenation (DO = 6.0 ± 0.1 mg/L) were analyzed by transcriptome sequencing (RNA-seq) and quantitative real-time PCR (qRT–PCR). RNA-seq was conducted on the muscle tissues of *E. coioides* in the hypoxia-tolerant (EMS), hypoxia-sensitive (EMW), and normoxic (CM) groups. Among the three groups, a total of 277 differentially expressed genes (DEGs) were identified. KEGG analysis revealed that the pathways significantly enriched after hypoxic stress are involved in the immune response, glycolysis/gluconeogenesis, energy metabolism, vasodilation and proliferation, cell proliferation, and apoptosis. qRT‒PCR verified that the differentially expressed genes *FIH-1, PHD-2, PPARα*, *BCL-XL*, *LDH-A*, and *Flt-1* were significantly upregulated after hypoxic stress and returned to normal levels after reoxygenation, suggesting that these DEGs play important roles in responding to hypoxia treatment. In addition, the HIF-1 signaling pathway was also activated under hypoxic stress, and qRT‒PCR confirmed that the expression level of *HIF-1α* was significantly elevated under acute hypoxic stress, indicating that the HIF-1 signaling pathway is the central pathway in the *E. coioides* hypoxic response mechanism and activates other related pathways to adapt to hypoxic stress. These pathways jointly regulate energy metabolism, substance synthesis, blood vessel proliferation, cell proliferation, and differentiation and prolong survival time. These results provide ideas for understanding physiological regulation after hypoxic stress and reoxygenation and provide basic insights for the future breeding of hypoxia-tolerant *E. coioides*.

## 1 Introduction

All organisms must deal with a variety of pressures that may affect their growth, health, and reproduction (Wilson, 2020). Oxygen is an important substrate for the normal biological processes of most organisms and is an essential factor to maintain metabolism and life activities (Childress and Seibel, 1998). Unlike that in the terrestrial environment, the dissolved oxygen (DO) level in the aquatic environment has a wide range of temporal and spatial variations (Ma et al., 2013). Hypoxia occurs when DO levels in aquatic environments drop below 2.0 mg/L and is accelerated by various factors, such as human activities, water pollution, and intensive fish farming (Vajner et al., 2006; Saetan et al., 2020; Sula et al., 2020). It has been well documented that hypoxia not only affects the survival and development of many aquatic species, but is also an important ecological factor leading to diseases (Cai et al., 2010; Wingfield, 2013). Aquaculture production declines sharply when there is no effective method to relieve or offset hypoxic stress (Dawood et al., 2021), which may also lead to huge economic losses and increasing declines in biodiversity and ecosystem function (Riedel et al., 2012; Villnäs et al., 2012).

Hypoxic stress is one of the most critical aquatic environmental factors that adversely affects the physiological processes of fish behavior, growth and development, reproduction, metabolism, and survival (Timmerman and Chapman, 2004; Papandreou et al., 2005; Pollock et al., 2007; Wang et al., 2020; Liu et al., 2021; Zheng et al., 2022). For example, hypoxia can reduce the fecundity of *Fundulus grandis* (Landry et al., 2007) and *Cyprinus carpio* (Wu et al., 2003) and inhibit the embryonic development of *Danio rerio* (Wang et al., 2020). In *Oreochromis niloticus*, acute hypoxia decreases the feeding activity and time, and growth rate (Zhou et al., 2001) and impairs the immune system (Gallage et al., 2016). Hypoxia adaptation is a complex process involving various physiological and biochemical processes, and molecular responses, such as hypoxia can increase erythrocyte number, reduce protein synthesis, enhance anaerobic metabolism regulate red blood cell proliferation, inhibit apoptosis, stimulate angiogenesis, and upregulate *HIF-1a* and other hypoxia-related gene expression (Carmeliet et al., 1998; Michiels, 2004; Terova et al., 2008; Muz et al., 2015; Lee et al., 2020). Under hypoxic conditions, glycogen content and lactate concentration decrease, and phosphoenolpyruvate carboxylate kinase expression increases in the liver of *Lateolabrax maculatus* (Yan et al., 2021).

*Epinephelus coioides* is the most economically important marine fish in China and other Asian countries (Føre et al., 2018; Yu et al., 2018). As this cultured fish is favored by consumers, its aquaculture industry is developing toward intensive modes such as cage farming and industrial farming to meet people’s demand for a higher yield. However, due to the natural environment and high nutrient input, high-density aquaculture usually leads to hypoxia, which restricts the cultivation of *E. coioides* (Wang et al., 2013; Huang et al., 2016). To date, few studies have uncovered the mechanism of the response to hypoxic stress in *E. coioides*.

As an important part of the fish body, muscle can maintain organism movement, anabolism, and catabolism, but its role can be affected by hypoxic stress (Liao et al., 2013). However, the hypoxia acclimation mechanism of muscle in *E. coioides* remains unknown. In this study, transcriptome sequencing (RNA-seq) was used to investigate the transcriptome profiles of the *E. coioides* muscle tissues among the hypoxia-tolerant (EMS), hypoxia-sensitive (EMW), and normoxic (CM) groups. Quantitative real-time PCR (qRT‒PCR) was used to analyze the expression sequences of related genes under hypoxic stress and reoxygenation according to the RNA-seq-screened hypoxia-related genes. Our results provide new insights for understanding the adaptation of fish to environmental stress and provide a theoretical basis for solving the problems of modern intensive aquaculture, especially high-density aquaculture.

## 2 Materials and methods

### 2.1 Fish acclimation

Healthy *E. coioides* (weight, 150 ± 5 g; length 15 ± 5 cm) were achieved from the Huizhou base of the Guangdong Marine Fisheries Experiment Center. These fish were acclimated in cylindrical tanks with constant ventilation for 2 weeks before prescription. The tanks were equipped with artificially disinfected self-circulating seawater with a salinity of 20–30 ppt and a temperature of 30 ± 1°C. The DO of the seawater was 6.0 ± 0.1 mg/L, as measured by a water quality analyzer (YSI, United States, ProQuatro). During acclimation, the fish were fed a commercial diet of 3% of their body weight daily. After adaptation, the fish were used for the hypoxic stress and reoxygenation experiment.

### 2.2 Hypoxic induction and sampling

Fifty-four individuals were kept in a breeding pool to conduct the pre-experiment. Briefly, the tank was sealed with a transparent plastic film, and nitrogen was continuously pumped through the nitrogen plastic ventilator until the fish were unbalanced (Pichavant et al., 2000; Li et al., 2018). At this moment, the water’s DO was found to be 0.6 ± 0.1 mg/L, which served as the benchmark for the next formal hypoxic stress test on *E. coioides*.

The experimental fish were split into two groups, the hypoxic group (H group) and the normoxic control group (CM group), with three replicates each and 54 fish for each replication. In the H group, the fish tanks were membrane-sealed and continuously nitrogen-injected to keep the DO level at 0.6 ± 0.1 mg/L. After 8 h of hypoxic stress, the reoxygenation (R) experiment was performed on the H group for 72 h, and the DO in the water was restored to 6.0 ± 0.1 mg/L. Throughout the hypoxia and reoxygenation experiment, the fish in the CM group tanks were continuously aerated to keep the DO at 6.0 ± 0.1 mg/L.

Sampling for RNA-seq began when half of the fish died after the hypoxic condition (DO 0.6 ± 0.1 mg/L) lasted for an hour in the H group. Following that, depending on their physiological states, the live fish were divided into the EMS and EMW group. Fish from each of the three groups (CM, EMS, EMW) were taken and dissected (three fish per replicate for a total of nine fish per group), and muscle tissues were taken and kept in liquid nitrogen.

Sampling for qRT‒PCR was performed when half of the fish were floating and unbalanced in the H group. Muscle tissues of the fish from the CM group and H group were collected at 0 h, 2 h, 4 h, 6 h, and 8 h after hypoxic stress. In the R group and CM group, muscle samples for qRT‒PCR were obtained at 0 h, 4 h, 8 h, 12 h, 24 h, 48 h, and 72 h after reoxygenation treatment. Muscle tissues were taken from 3 fish in each parallel group at each interval, and placed at −80°C for further analysis.

### 2.3 RNA extraction, library creation, and sequencing

Following the manufacturer’s instructions, total RNA from the muscle was extracted using TRIzol kits (Promega, Madison, WI, United States). The quantity and quality of the RNA were determined using a Bioanalyzer 2100 (Agilent Technologies, United States), ND-2000 (NanoDrop Technologies, United States), and RNase-free agarose gel electrophoresis, respectively. High-quality RNA (RIN> 7) was applied to create the sequencing libraries. Three biological replicate samples were set up in each group. Finally, nine cDNA libraries (CM-3, EMS-3, EMW-3) were scanned using paired-end technology (Guangzhou Gene Jirui Gene Technology Co., Ltd., Guangzhou, China) around an Illumina HiSeq 2500 sequencing (Illumina, San Diego, CA, United States).

### 2.4 Sequence concatenation and gene annotation

After CASAVA base calling, the raw data from the Illumina NovaSeq 6000 sequencing machine were transformed into text data and stored in FASTQ format as raw reads. Fastp (v 0.12) was used to remove low-quality sequences from the raw reads (Altschul et al., 1997). The effectiveness of RNA-sequencing was assessed by the quality scores of the Q20, Q30, and GC contents. The clean data were aligned to the *E. coioides* genome utilizing TOPHAT2 (v.2.0.4). The relative expression abundance of unigenes was determined as the reads per kb per million reads (RPKM).

### 2.5 Identification and enrichment analysis of differential gene expression

DESeq2 was applied to discover differentially expressed genes (DEGs) among the three groups. Absolute log2 fold change |log_2_FC| > 1 and false discovery rate (FDR) < 0.05 were defined as DEGs. The DEGs were subjected to GO functional annotation and pathway enrichment analysis *via* GOATOOLS (v. 0.6.5) (Ye et al., 2018) and KOBAS v.2.1 (Wu et al., 2006), respectively. Enrichment analysis of the KEGG pathway provided functional annotations of the DEGs. An FDR-adjusted *p* value ≤0.05 was used to determine the significantly enriched GO terms or pathways. DEGs implicated in metabolism, cellular processes, environmental information processing, organismal systems, human diseases, and genetic information processing were screened for further investigation to clarify the physiological changes in *E. coioides* under hypoxia.

### 2.6 qRT‒PCR validation for DEGs

qRT‒PCR of the selected DEGs was performed to evaluate the reliability of the RNA-seq results. The specific primers shown in Supplementary Table S1 for the chosen DEGs were created through Primer v.6.0. qRT‒PCR was carried out on a 384-well plate qRT‒PCR device (ABI 7500; Applied Biosystems, Waltham, MA, United States) with a final volume of 12.5 μL including 6.25 μL of SYBR qRT‒PCR Master Mix (Novizan, China), 4.25 μL of ddH_2_O, 0.5 μL of each primer (the primers used for qRT‒PCR are displayed in Supplementary Table S1), and 1 μL of template DNA (50 ng/μL). The qRT‒PCR conditions were as follows: 95°C for 5 min; 40 cycles of 95°C for 5 s, 60°C for 30 s, and 72°C for 30 s; and 72°C for 10 min for data collection. Each sample was examined in triplicate for this experiment. The gene expression levels were determined by the 2^−ΔΔCT^ method with *β*-actin as the internal reference. Statistical analyses of the experimental data were carried out by SPSS (v 20.0). The results are presented as the mean ± SE and were graphed using Origin 9.0 software (Zhong et al., 2021).

### 2.7 Detection of hypoxic stress and reoxygenation-related genes

Total RNA from each sample was extracted by RNA isolater Total RNA Extraction Reagent following the manufacturer’s instructions. RNA concentration and purity were measured on a spectrophotometer. First-strand cDNA was synthesized according to the instructions of the HiScript^®^ II Q RT SuperMix for qPCR (+gDNA wiper) reverse transcription kit and then stored at -80°C for further study. The primers for qRT‒PCR detection of hypoxic stress- and reoxygenation-related genes in muscle tissue of *E. coioides* are shown in Supplementary Table S2. The qRT‒PCR conditions and protocol were the same as those described in Section 2.6.

## 3 Results

### 3.1 Summary of RNA-seq data

Transcriptomic sequencing was performed from the muscle of *E. coioides*. After mass filtration, a total of 45,597,702 raw reads (68.83 Gb) were obtained, of which 45,313,712 passed the quality control process. The size of the clean reads in each library ranged from 6.50 Gb to 8.99 Gb. The clean reads were compared with the reference genome, and the statistical parameters of clean reads in the CM, EMS, and EMW groups were as follows: Q20, 97.46–97.92%; Q30, 93.41–94.36%; GC content, 51.01–51.89%; and error rate, 0.025–0.026%. Clean reads from each library were sequentially mapped with reference genomic information from *Epinephelus* spp., with a total ratio of 87.60%–89.71% per sample, and more than 5.75 Gb of data for each library was obtained (Table 1).

**TABLE 1:** Overview of the RNA-seq library of *E. coioides*.

Sample	Raw reads	Clean reads	Clean bases (Gb)	Q20%	Q30%	GC%	Error%	Total mapped rate (%)
CM1	60,390,328	60,333,588	8.99	97.92	94.36	51.87	0.025	88.90
CM2	43,703,130	43,667,170	6.5	97.75	94.08	51.42	0.025	88.55
CM3	51,377,400	51,315,348	7.66	97.53	93.53	51.89	0.026	89.55
EMS1	55,037,718	55,011,260	8.19	97.8	94.22	51.1	0.025	88.22
EMS2	44,984,404	44,964,612	6.7	97.88	94.3	51.57	0.025	88.92
EMS3	53,574,682	53,542,472	7.97	97.86	94.27	51.59	0.025	87.60
EMW1	46,250,658	46,230,238	6.89	97.55	93.61	51.28	0.026	89.06
EMW2	49,270,864	49,254,648	7.35	97.46	93.41	51.01	0.026	88.95
EMW3	49,008,518	48,994,376	7.31	97.7	93.99	51.44	0.025	89.71

C: normoxic control group, M: muscle tissue, S: hypoxia-tolerant group, W: hypoxia-sensitive group.

### 3.2 Screening of DEGs between groups

**FIGURE 1:**
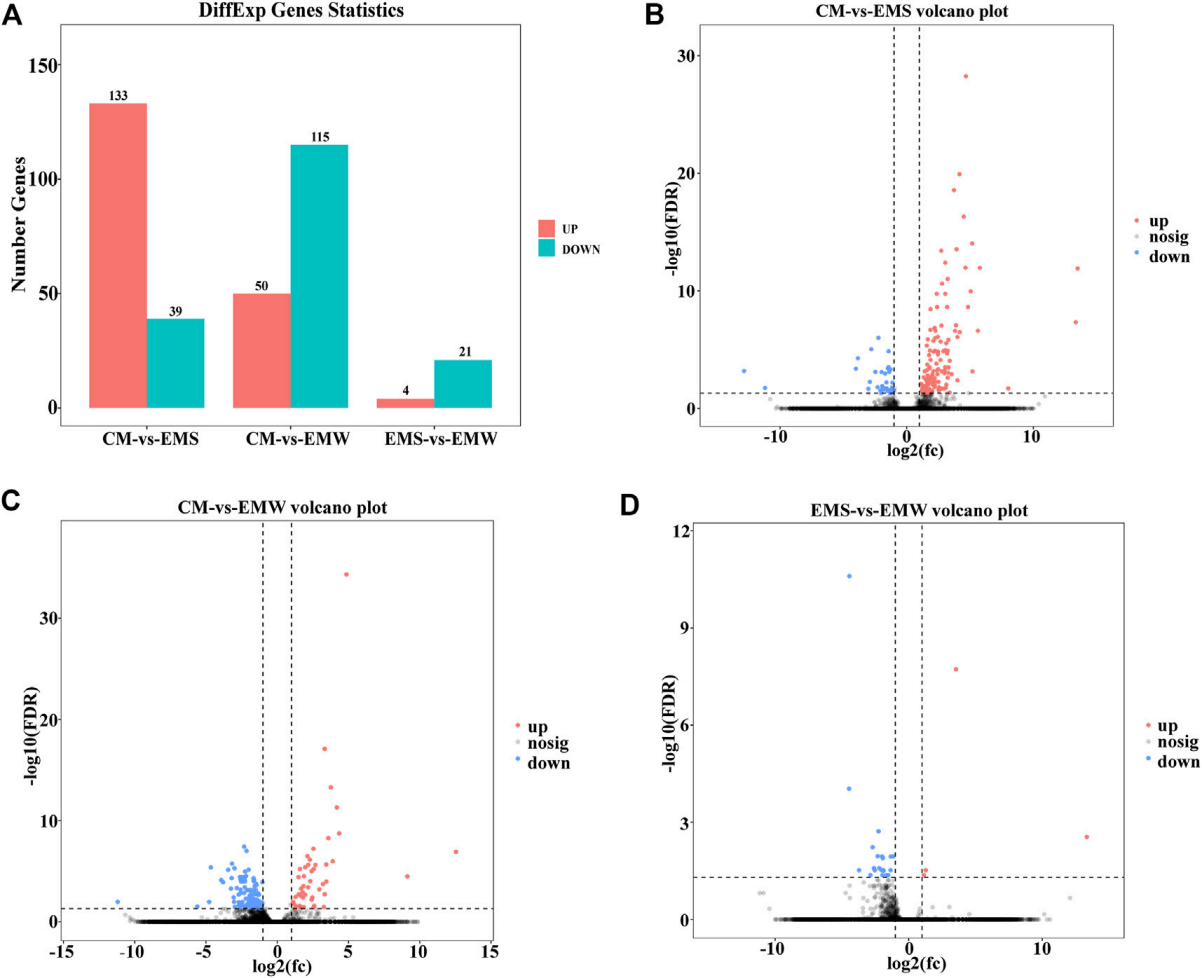
Differences in gene number in CM vs. EMS, CM vs. EMW, and EMS vs. EMW groups. **(A)** The bar chart shows the number of genes in the CM vs. EMS, CM vs. EMW, and EMS vs. EMW groups. **(B)** Volcano plot of DEGs in CM vs. EMS. **(C)** Volcano plot of DEGs in CM vs. EMW. **(D)** Volcano plot of EMS vs. EMW.

As shown in Supplementary Figure S1, 277 DEGs were found in the CM vs. EMS, CM vs. EMW, and EMS vs. EMW groups (Supplementary Figure S1). In the CM vs. EMS groups, a total of 172 DEGs were identified, with 133 upregulated DEGs and 39 downregulated DEGs (Figures 1A,B). The comparison of CM and EMW displayed 165 DEGs, of which 50 were upregulated and 115 were downregulated (Figures 1A,C). There were only 25 DEGs between the EMS and EMW groups, with 4 DEGs upregulated and 21 DEGs downregulated (Figures 1A,D).

### 3.3 GO enrichment analysis of DEGs

**FIGURE 2:**
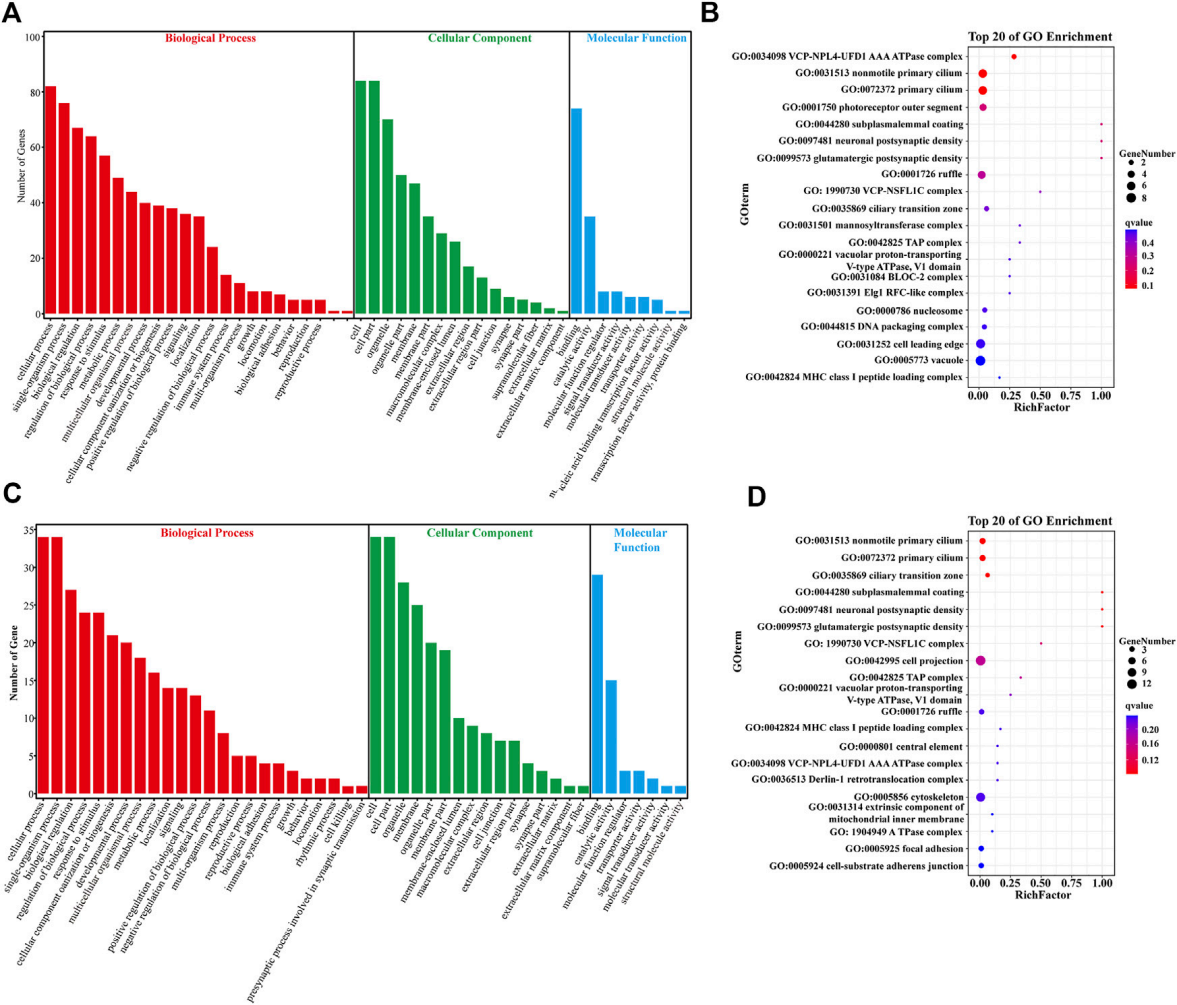
Gene ontology (GO) analysis of DEGs. **(A)** GO pathway analysis of DEGs in the CM vs. EMS groups. **(B)** Top 20 enriched terms by DEGs in the CM vs. EMS groups. **(C)** GO pathway analysis of DEGs in the CM vs. EMW groups. **(D)** Top 20 enriched terms by DEGs in the CM vs. EMW groups. The color of each dot represents the corrected *p*-value of the corresponding item.

In the CM vs. EMS comparison, 172 DEGs were screened for GO enrichment analysis and functional classification, and the results showed that they were divided into three main functional categories, namely, the biological process (BP), cellular component (CC), and molecular function (MF) categories. The DEGs were mainly associated with BP terms such as the cellular process, tissue process, biological regulation, stimulus-response and metabolic process terms (Figure 2A). To further elucidate the biological events of DEGs primarily involved in the hypoxia response, the top 20 most enriched GO terms were listed (Figure 2B). The DEGs were mainly enriched in energy metabolism terms (GO:0034098 VCP-NPL4-UFD1 AAA ATPase complex), cilium-related terms (GO:0072372 primary cilium, GO:0031513 nonmotile primary cilium), and other BP terms (Figure 2B).

In the CM vs. EMW comparison, GO enrichment analysis revealed that the165 screened DEGs were also divided into BP, CC, and MF groups, and the DEGs were mainly related to BP terms (Figure 2C). To further elucidate the biological events of DEGs primarily participated in the hypoxia response, the 20 most enriched GO terms were listed, which were mainly associated with BP terms such as cytoskeleton movement (GO:0005856 cytoskeleton), cilia movement (GO:0072372 primary cilium), and synthesis of ATPase complex (Figure 2D).

### 3.4 KEGG analysis of DEGs

**FIGURE 3:**
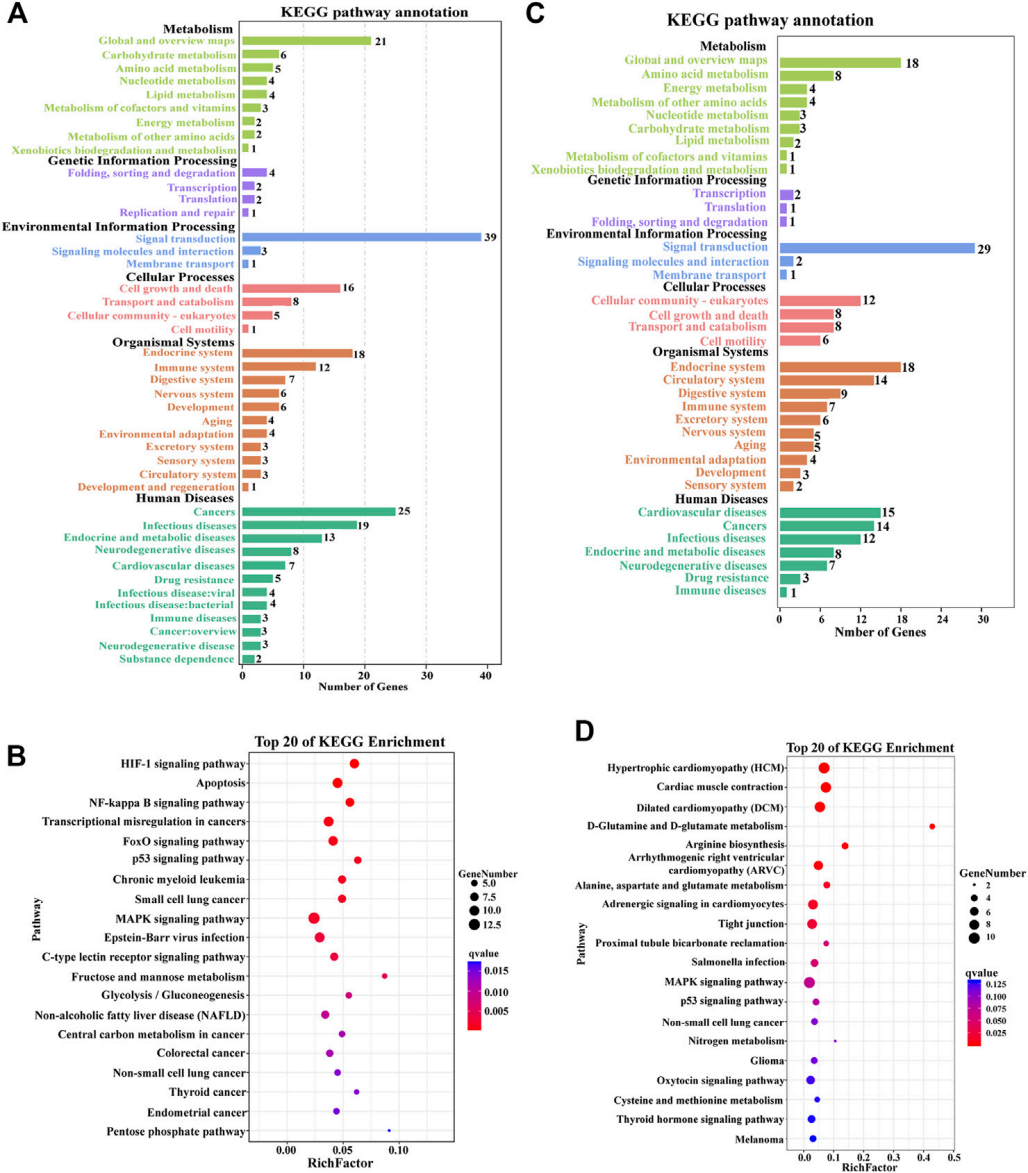
Kyoto Encyclopedia of Genes and Genomes (KEGG) pathway analysis of DEGs in the muscle of *E. coioides*. **(A)** KEGG pathway enrichment analysis of DEGs in the CM vs. EMS groups. **(B)** Top 20 enriched pathways by DEGs in the CM vs. EMS groups. **(C)** KEGG pathway enrichment analysis of DEGs in the CM vs. EMW groups. **(D)** Top 20 enriched pathways by DEGs in the CM vs. EMW groups.

KEGG analysis showed that a total of 43 pathways were enriched between the CM group and EMS group, classified into six functional categories: metabolism, environmental information processing, organic systems, human diseases, and cellular processes (Figure 3A). Signal transduction, global and overview maps, carbohydrate metabolism, and cell growth and death were the dominant pathways of enrichment (Figure 3A). All DEGs obtained in CM vs. EMS were mapped to KEGG to identify specific biological pathways of the hypoxia response. The top 20 most abundant KEGG pathways associated with the hypoxia mechanism were the HIF-1 signaling pathway, apoptosis, NF-kappa B signaling pathway, FOXO signaling pathway, MAPK signaling pathway, C-Type Lectin receptor signaling pathway, and glycolysis/gluconeogenesis (Figure 3B). The above-enriched pathways were mainly involved in the immune response, cell proliferation, apoptosis, and energy metabolism.

In the comparison of the CM and EMW groups, 36 significantly enriched KEGG annotations were found, consisting of 6 different functional categories. These pathways were mainly related to signal transduction, fatty acid metabolism, cell growth and death, the endocrine system, and the circulatory system (Figure 3C). Among the 20 most enriched KEGG pathways, the MAPK signaling pathway, adrenergic signaling in cardiomyocytes, and cardiac muscle contraction were significantly enriched and involved in the hypoxia response (Figure 3D).

### 3.5 Protein‒protein interaction (PPI) network analysis of the major DEGs

**FIGURE 4:**
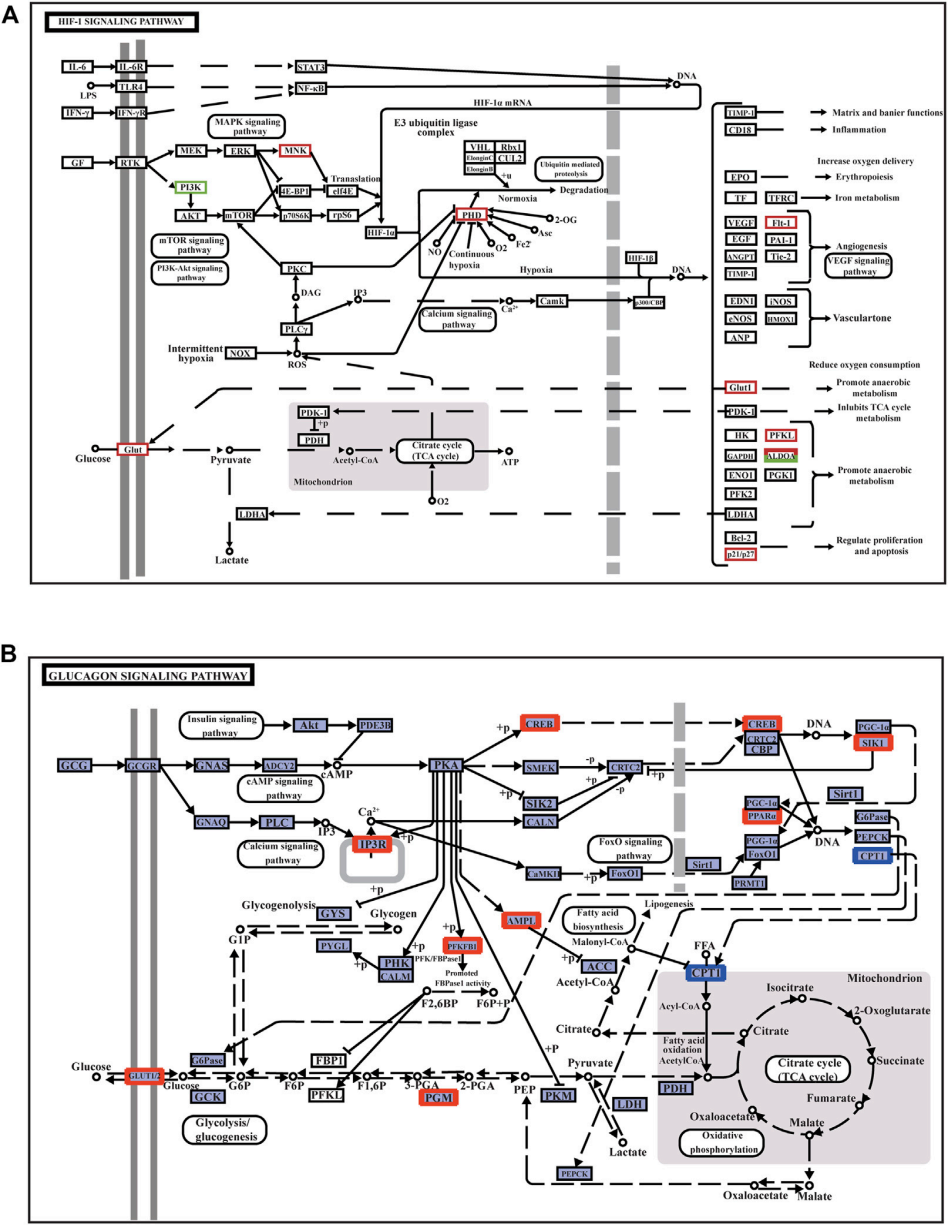
Annotation diagram of muscle signaling pathway and its related DEGs. **(A)** HIF-1 signaling pathway. **(B)** Glucagon signaling pathway. The red box indicates the upregulated genes in the pathways; the green box represents the downregulated genes in the pathway.

PPI network analysis showed that in the EMS group, the DEGs were mainly involved in the HIF-1 signaling pathway, FOXO signaling pathway, AMPK signaling pathway, MAPK signaling pathway, NF-κB signaling pathway, vascular endothelial growth factor (VEGF) signaling pathway, cAMP signaling pathway, and glycolysis/gluconeogenesis signaling pathway (Figures 4A,B), while there was no significant difference in the EMW group.

The HIF-1 signaling pathway is the main pathway activated when muscle cells are damaged by hypoxic stress. In the HIF-1 signaling pathway, the MNK factor in the MAPK signaling pathway is upregulated; thus, *HIF-1α* mRNA expression is upregulated. The related gene *VEGF receptor-1* (*Flt-1*) is upregulated to trigger the VEGF signaling pathway, promote angiogenesis and proliferation, and accelerate blood oxygen delivery to adjust to a hypoxic environment. Upregulation of cyclin p21/p27 regulates cell proliferation and reduces apoptosis (Figure 4A). The upregulation of *phosphofructokinase-1* (*PFKL*) and recombinant human fructose-bisphosphate aldolase A (ALDOA) promotes anaerobic metabolism and reduces oxygen consumption. Glucose transporter 1 (Glut-1) is involved in glycolysis/gluconeogenesis in response to hypoxia to enhance glucose transport and utilization (Figure 4B). The glycolysis/gluconeogenesis pathway is regulated by the FOXO signaling pathway and the calcium signaling pathway (Supplementary Figure S2). In the FOXO signaling pathway, cyclin p27 is an important member of the cyclin-dependent inhibitor CIP/KIP family. P27 and growth arrest- and DNA damage-inducible 45 (Gadd45), which function to regulate the cell cycle, were also upregulated when muscle tissue was damaged under hypoxic stress. Gadd45 can also regulate antioxidant stress and DNA repair in the hypoxia response (Supplementary Figure S2).

### 3.6 Key DEGs involved in the hypoxia response

**FIGURE 5:**
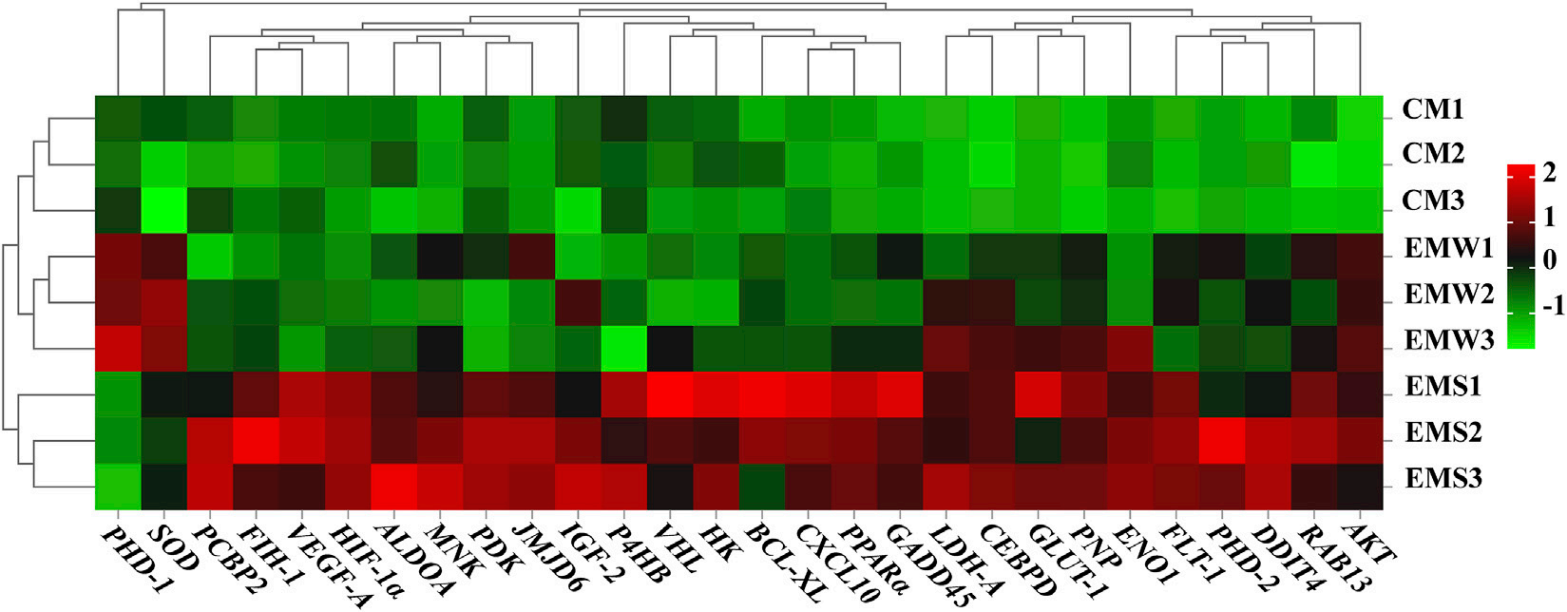
Heat map of DEGs in the control and experimental groups. Red represents gene up-regulation, green represents gene down-regulation. CM indicates the normoxic control group of muscle tissue; EMS represents hypoxia-tolerant group of muscle tissue; EMW represents hypoxia-sensitive group of muscle tissue.

To comprehend the mechanism underlying *E. coioides’s* adaptive reaction to hypoxia, the DEGs associated with hypoxia were further screened. As shown in Figure 5, 26 DEGs were discovered to be significantly upregulated in the EMS group, while in the EMW group, only 2 DEGs, *proline hydroxylase-1* (*PHD-1*) and *superoxide dismutase* (*SOD*), were detected as significantly upregulated. As expected, none of these 28 DEGs were found in the CM group (Figure 5). The screened DEGs in the EMS group were divided into two categories. One group included upstream pathway DEGs, including *HIF-1α*, *factor inhibiting HIF-1* (*FIH-1*), *PHD-2*, *protein kinase* (*AKT*), *MAPK-activated kinase* (*MNK*) and *von Hipel-Lindau protein* (*VHL*). Another group of downstream pathway DEGs included genes associated with energy metabolism, such as *pyruvate dehydrogenase kinase* (*PDK*), l*actate dehydrogenase A* (*LDH-A*, *GLUT-1*, *ALDOA*, and *hexokinase* (*HK*), and genes involved in regulating cell proliferation and apoptosis, including poly *(rC) binding protein 2* (*PCBP2*), DNA damage-inducible transcript 4 (*DDIT4*), *CCAAT*/enhancer-binding protein delta (*CEBPD*), Ras-related protein Rab-13 (*RAB13*), purine nucleoside phosphorylase (*PNP*), *C-X-C* motif chemokine 10 (*CXCL10*), peroxisome proliferator-activated receptor-α (*PPARα*), B-cell lymphoma-extra-large (*BCL-XL*) and *GADD45*. In addition, the downstream pathway DEGs also included *VEGF-A*, *Flt-1*, and *enolase* (*EN O 1*), which were related to the *VEGF-A* pathway, proliferation, and anaerobic metabolism. The activation of these genes is involved in the major biological processes that participate in the response to hypoxic stress (Figure 5).

### 3.7 qRT‒PCR validation

**FIGURE 6:**
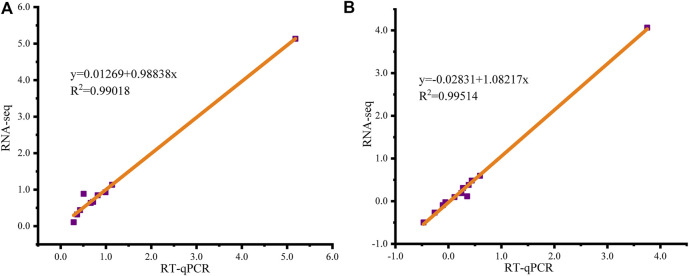
Fitting curve of correlation between qRT‒PCR and RNA-seq gene expression results. X-axis is the log_2_FC value of qRT‒PCR, Y-axis is the log_2_FC value of RNA-seq, and the red line is the fitting curve. **(A)** Correlation analysis of EMS group. **(B)** Correlation analysis of EMW group.

To confirm the accuracy of the RNA-seq data, the transcript levels of *HIF-1α*, *PHD-2*, *LDH-A*, *VHL*, *VEGF-A*, *FIH-1*, *insulin-like growth factor-2* (*IGF-2*), *SOD*, *HK*, and *pyruvate kinase* (*PK*) in the EMS group and those of *HIF-1α*, *PHD-2*, *LDH-A*, *VHL*, *VEGF-A*, *PHD-1*, *FIH-1*, *IGF-2*, *SOD*, *catalase* (*CAT*), *HK* and *PK* in the EMW group were analyzed and verified by qRT‒PCR. Correlation analysis was performed on the obtained results using the log_2_FC values. The fitting curve equation of the EMS group was y = 0.01269 + 0.98838x, R^2^ = 0.99018 (Figure 6A), while that of the EMW group was y = 0.02831 + 1.08217x, R^2^ = 0.99514 (Figure 6B). The EMS and EMW groups’ R^2^ values were both greater than 0.7, demonstrating the high accuracy of the data from transcriptome sequencing.

### 3.8 Expression analysis of seven key DEGs after hypoxia and reoxygenation

**FIGURE 7:**
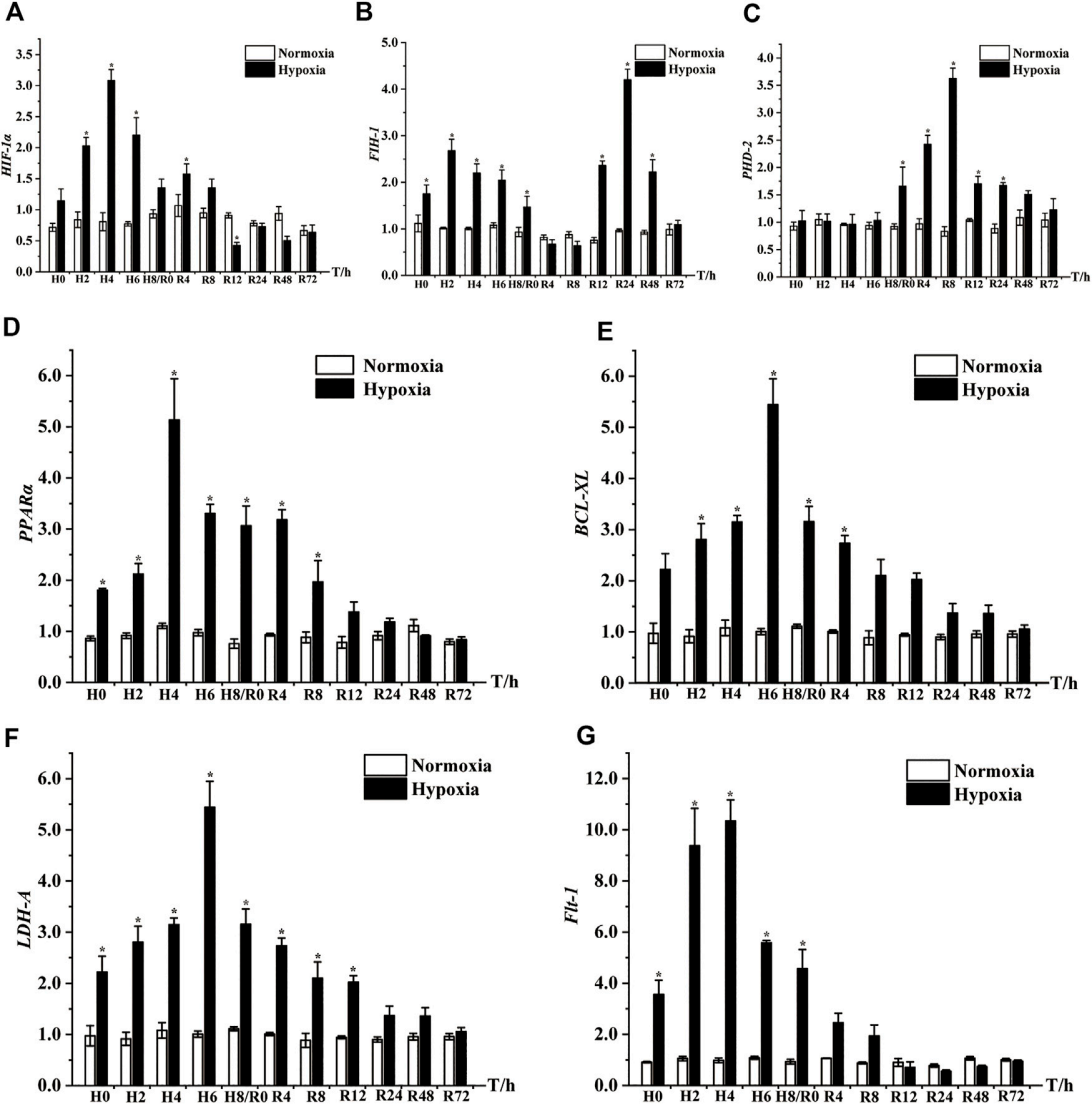
Expression patterns of seven key DEGs in muscle of *E. coioides* after hypoxic stress and reoxygenation, including *HIF-1α* **(A)**, *FIH-1* **(B)**, *PHD-2* **(C)** *, PPARα* **(D)** *, BCL-XL* **(E)** *, LDH-A* **(F)**, *Flt-1* **(G)**. H: Hypoxia; R: Reoxygenation. The results were represented by mean and standard error, and the difference of labeled lowercase letters indicated significant difference (*p < 0.05*).

qRT‒PCR was carried out to illustrate the mRNA expression levels of seven significant DEGs in the muscle after hypoxia and reoxygenation treatment. As shown in Figure 7, the expression levels of *HIF-1α*, *FIH-1*, *PPARα*, *BCL-XL*, *LDH-A* and *Flt-1* were remarkably upregulated at all time points after acute hypoxic stress compared with normoxic controls, peaking at 4 h of hypoxia (H4), H2, H4, H6, H6, and H4, respectively. However, the expression level of *PHD-2* was significantly increased only at H8. After the restoration of dissolved oxygen, the expression of these genes successively returned to levels similar to those of normoxic controls. The expression of both *HIF-1α* and *LDH-A* recovered to normal control levels at 24 h of reoxygenation (R24), *Flt-1*, *PPARα* and *BCL-XL* all returned to normal levels at R12, while *PHD-2* and *FIH-1* returned to normal expression levels at R48 and R72, respectively (Figure 7).

## 4 Discussion

As we all know, hypoxia is a major problem in high-density fish aquaculture. Therefore, screening for candidate genes associated with hypoxia tolerance traits is extremely important for the selection and breeding of hypoxia-tolerant species in intensive fish farming. To provide a theoretical basis for solving the problems of modern intensive aquaculture of *E. coioides*, the main aim of this paper is to identify underlying candidate genes related to hypoxia tolerance. In the present study, RNA-seq was performed on the EMS, EMW and CM individuals, and qRT‒PCR was adopted to analyze the expression level of DEGs screened from the RNA-seq based on the samples of hypoxic stress and reoxygenation. Fortunately, some significantly enriched pathways and DEGs were identified from the hypoxia-tolerant group by RNA-seq and qRT‒PCR analyses.

In the current study, KEGG analysis of DEGs showed that the upstream HIF-1 signaling pathway was enriched in the EMS group, while HIF-1α or *HIF-1α* mRNA expression was upregulated in the HIF-1 signaling pathway annotation. Previous studies have shown that the HIF-1 signaling pathway is the central pathway in the response to hypoxia (Sheng et al., 2017) and that it can be activated quickly after hypoxic stress to enable adaptation to a hypoxic environment. As a critical regulator in the HIF-1 signaling pathway, the *HIF-1α* gene plays an important role in the homeostatic regulation of organisms (Minet et al., 1999). Our results showed that *HIF-1α* expression was dramatically elevated under acute hypoxic stress but returned to normal after reoxygenation, which is similar to findings in *Lateolabrax japonicus* (Terova et al., 2008), implying that *HIF-1α* plays a key role in the response to hypoxic stress. These results suggest that the HIF-1 signaling pathway is the core of the molecular regulatory pathway that is activated during hypoxia and reoxygenation of *E. coioides* and triggers a series of metabolic changes, including changes in energy metabolism, the immune response, and other metabolic pathways.

*Flt*-1 can stimulate the VEGF signaling pathway and promote vascular dilation and proliferation (Jiang et al., 1997; Shan et al., 2012). qRT‒PCR analysis on *Flt-1* showed significant upregulation under hypoxic stress, which was consistent with the transcriptome sequencing results. Both the *PHD-2* and *FIH-1* genes can theoretically inhibit *HIF-1α* expression (Grandcourt et al., 2009; Jelkmann, 2011; Wang et al., 2015). In this study, similar to that of *HIF-1α,* the expression of *FIH-1* was significantly increased under hypoxic stress in the H0-H8 stage, unlike the expression of *PHD-2,* which did not differ significantly from that of the control group. After 12 h of reoxygenation, *PHD-2* and *FIH-*1 expression levels were significantly increased compared with those of the normoxic group, while the *HIF-1α* level decreased significantly until the R24 period (Figure 7). These results suggest that *PHD-2* and *FIH-1* are inhibitory factors of *HIF-1α* expression within 8–24 h after reoxygenation. Unexpectedly, PHD-2 expression peaked at R8 point in the reoxygenation stage, we speculated that there were two possible reasons for this. On the one hand, species variability leads to a different expression pattern of *E. coioides PHD-2* in the reoxygenation stage than that in other fish, on the other hand, *PHD-2* may participate in down-regulating the expression of *HIF-1α* and other genes during the reoxygenation phase, which needs further study in the future.

During hypoxic stress, extracellular signaling factors specifically bind to intracellular or cell membrane receptors to induce intracellular signals and trigger cascade reactions, thus affecting cell functions (Chen et al., 2001; Sauer et al., 2001). The HIF-1 signaling pathway coordinates the repair of inflammation by activating the NF-κB signaling pathway (Spirina et al., 2017; Mohamed et al., 2019). The AMPK and FOXO signaling pathways complete cell proliferation and differentiation, complete the distribution and reorganization of intracellular energy metabolism, and repair the inflammation caused by metabolic regulation (Nagata et al., 2003; Lee et al., 2013; Dengler, 2020). Our results showed that oxidative phosphorylation, the NF-κB signaling pathway, the FOXO signaling pathway, the p53 signaling pathway, the MAPK signaling pathway, and apoptosis were highly enriched in the EMS group and that DEGs in these pathways were significantly upregulated. For example, *PPARα* and *BCL-XL* are involved in the immune response process to repair inflammatory damage, slow apoptosis, and prolong survival time. The qRT‒PCR results showed that *PPARα* and *BCL-XL* were also significantly upregulated under hypoxic stress (Figure 7), indicating that *PPARα* and *BCL-XL* can repair muscle tissue inflammatory damage caused by hypoxic stress.

Activation of anaerobic metabolism is related to hypoxic stress. Under hypoxic stress, fish respiration changes from aerobic to anaerobic respiration (Maccormack et al., 2006). Anaerobic glycolysis/glucose metabolism is the cause of the high energy demand of fish under hypoxic stress. Glycolysis is an important energy-providing pathway during hypoxic stress (Connett et al., 1990; Semenza et al., 1994; Xie et al., 2019; Kierans and Taylor, 2021). Here, the glycolysis/glucose metabolism signaling pathway was also highly enriched in the EMS group. As a crucial REDOX enzyme in the glycolysis pathway, *LDH-A* can reversely catalyze the conversion of lactic acid to pyruvate and provide ATP and prevent acidosis caused by excess lactic acid (Shim et al., 1997; Fantin et al., 2006). We found that *LDH-A* expression was also significantly elevated under acute hypoxia treatment but returned to normal under reoxygenation, in line with the outcomes shown in *Oncorhynchus mykiss* (Davie et al., 1986). These results suggest that glycolysis and glucose metabolism are of great significance in physiological adaptation and the response to acute hypoxic stress and reoxygenation.

## 5 Conclusion

The mechanism of the *E. coioides* muscle response to acute hypoxia and reoxygenation was investigated by RNA-seq and qRT‒PCR in this study. 277 DEGs were identified, and several key DEGs were verified by qRT‒PCR to be significantly upregulated after hypoxic stress but restored to normal levels after reoxygenation. The significantly enriched pathways after hypoxia induction were involved in immune processes, energy metabolism, apoptosis and so on. These results are beneficial for understanding adaptability to hypoxic environments and provide valuable information for the breeding of *E. coioides* and other fish with low oxygen tolerance.

## Data Availability

The datasets presented in this study can be found in online repositories. The names of the repository/repositories and accession number(s) are below: NCBI (accession: PRJNA895010).
