# Supplementary material for: RNA-seq and qRT-PCR analyses reveal the physiological response to acute hypoxia and reoxygenation in Epinephelus coioides
Source: Front Physiol. 2022 Nov 3;13:1049776. doi: 10.3389/fphys.2022.1049776 (PMC9670319; doi:10.3389/fphys.2022.1049776)
Supplement: Supplementary file 1 [file DataSheet1.docx]

***Supplementary Material***

**Supplementary Table 1.** Primers for RT-qPCR of muscle tissue of *E. coioides*.

**Supplementary Table 2.** Primers for qPCR detection of hypoxia stress and reoxygenation-related genes in muscle tissue of *E. coioides*.

**Supplementary Figure 1.** Venn diagrams of DEGs in various group combinations: CM vs. EMS, CM vs. EMW, and EMS vs. EMW.

**Supplementary Figure 2.** Annotation diagram of muscle signaling pathway and its related DEGs. FOXO signaling pathway. The red box indicates the upregulated genes in the pathways; the green box represents the downregulated genes in the pathway.

**Table S1**

| Gene Name | Forward Primer | Reverse Primer |
| --- | --- | --- |
| *β-actin* | TACGAGCTGCCTGACGGACA | GGCTGTGATCTCCTTCTGC |
| *HIF-1α* | AGCTCATGGGTTATGATCCAGAC | TTGTTGTAGATGACAGTGGCTTG |
| *PHD-2* | AAGGCGACTCCACTAAAGACATCC | TCCATTTATCGTGTAGTTTCCCAGT |
| *LDH-A* | CCTAATCCGTACACTCCTTGTCT | AGGTACTATCACTGGATTGTGGC |
| *VEGF-A* | CGAAGCGATGGAGTGCGTACC | GCAGGAGCATTTACAGGTGAGAGG |
| *VHL* | ATTTGAGCAGCTTTGGTTGC | GGCTAAGACTTCCTCTGCTG |
| *FIH-1* | CCAAGTCGCAACGGACGGAA | TGGCTTGCTGCTTGTTGATCC |
| *IGF-2* | CCTGAAGGCTGATGAGCTGTGC | TTGCGGATGGCGACGAGTTTG |
| *SOD* | GCGGACAGGCATGTTGGAGAC | CCAATGACACCACAGGCTAGACG |
| *PHD-1* | ACCATCGTAGACTCAGGCAAAAGC | ATCGCCCTCTTTCTTGTGTTGTCG |
| *CAT* | CTATTGCTGTCCGCTTCTCCACTG | TGAAGGTGTGAGAGCCGTAGCC |
| *HK* | AGAGGTACGGAGGAGAGGAGGAG | GGTGCCAGTGCCAACGATGAG |
| *PK* | GTCCGCACACAGCACAAGGTAG | AGTTCACACGCAGGTCAACATCC |

**Table S2**

| Gene Name | Forward Primer | Reverse Primer |
| --- | --- | --- |
| *β-actin* | TACGAGCTGCCTGACGGACA | GGCTGTGATCTCCTTCTGC |
| *HIF-1α* | GTGAAGCCAGTGAAGGAGGAACAG | TGGTGGTGGTGACATCGAGAGG |
| *FIH-1* | CCAAGTCGCAACGGACGGAA | TGGCTTGCTGCTTGTTGATCC |
| *PHD-2* | AAGGCGACTCCACTAAAGACATCC | TCCATTTATCGTGTAGTTTCCCAGT |
| *LDH-A* | CCTAATCCGTACACTCCTTGTCT | AGGTACTATCACTGGATTGTGGC |
| *PPARα* | TAAAGCTGAAGGCCGAGATG | ATGTCGTGGATGACGAAAGG |
| *BCL-XL* | AGCTGGTTTCCCGCATCACA | CGACCAGCACTCCCATGAGC |
| *FLT-1* | CGGGATGTGGCAGAGGAGGA | TCCTGCAACGACACCGCTTT |

**Figure S1**


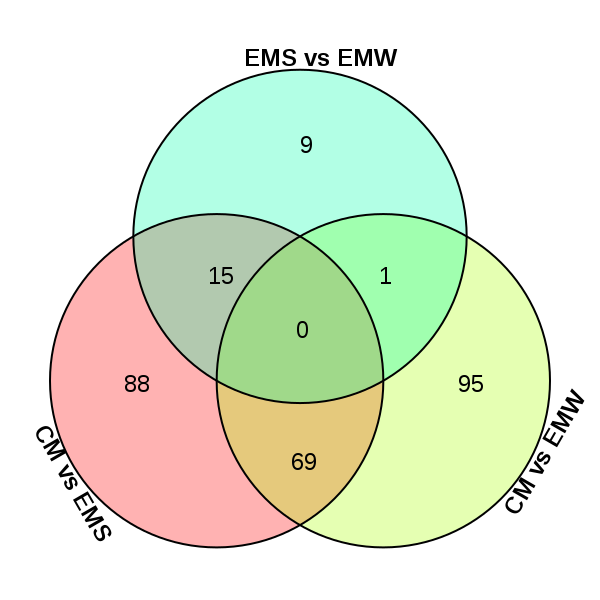


**Figure S2**

**
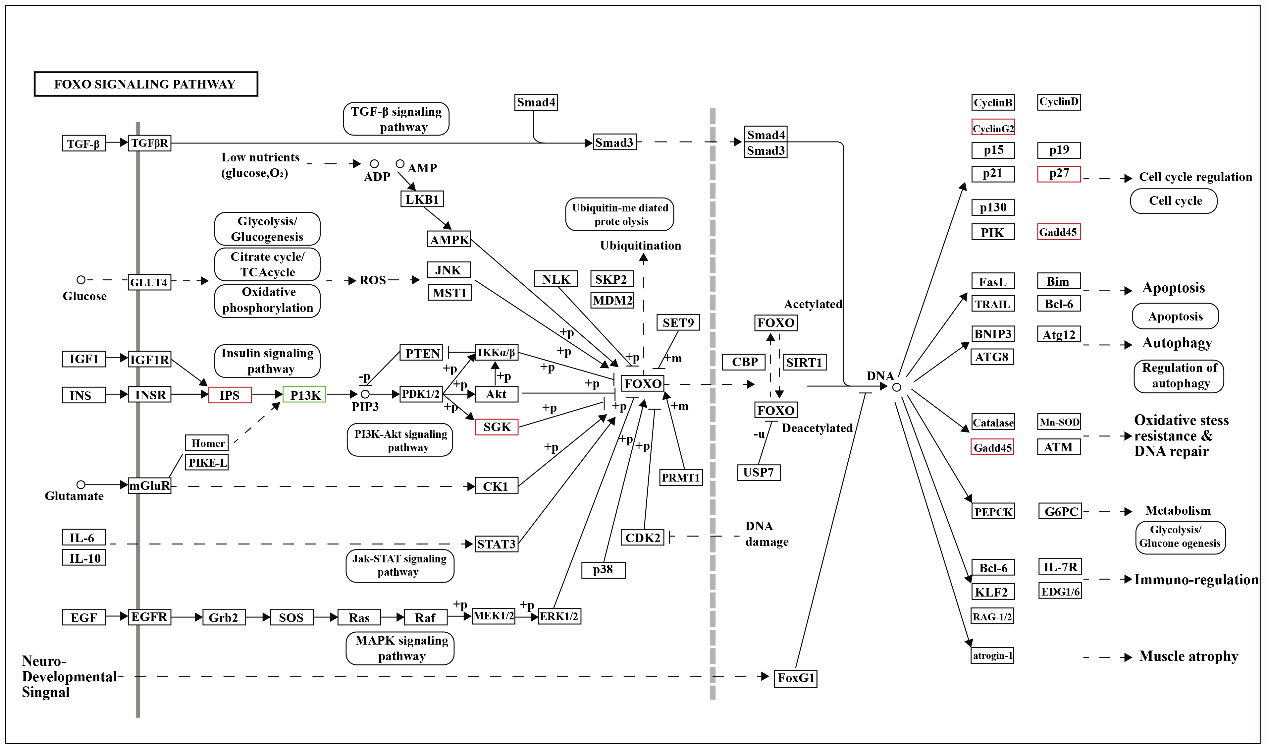
**
